# Diagnosis of tuberculosis in wildlife: a systematic review

**DOI:** 10.1186/s13567-020-00881-y

**Published:** 2021-02-24

**Authors:** Jobin Thomas, Ana Balseiro, Christian Gortázar, María A. Risalde

**Affiliations:** 1 Sanidad Y Biotecnología (SaBio), Instituto de Investigación en Recursos Cinegéticos IREC (UCLM-CSIC), 13003 Ciudad Real, Spain; 2 Indian Council of Agricultural Research (ICAR), New Delhi, 110001 India; 3 Departamento de Sanidad Animal, Facultad de Veterinaria, Universidad de León, 24071 León, Spain; 4 Departamento de Sanidad Animal, Instituto de Ganadería de Montaña (CSIC-Universidad de León), Finca Marzanas, Grulleros, 24346 León, Spain; 5 Departamento de Anatomía Y Anatomía Patológica Comparadas Y Toxicología. Facultad de Veterinaria, Universidad de Córdoba (UCO), 14014 Córdoba, Spain; 6 Unidad de Enfermedades Infecciosas, Grupo de Virología Clínica Y Zoonosis, Instituto Maimónides de Investigación Biomédica de Córdoba (IMIBIC), Hospital Reina Sofía, Universidad de Córdoba (UCO), 14004 Córdoba, Spain

**Keywords:** animal tuberculosis, diagnosis, immunological methods, *Mycobacterium tuberculosis* complex, PRISMA, systematic review, wildlife

## Abstract

Animal tuberculosis (TB) is a multi-host disease caused by members of the *Mycobacterium tuberculosis* complex (MTC). Due to its impact on economy, sanitary standards of milk and meat industry, public health and conservation, TB control is an actively ongoing research subject. Several wildlife species are involved in the maintenance and transmission of TB, so that new approaches to wildlife TB diagnosis have gained relevance in recent years. Diagnosis is a paramount step for screening, epidemiological investigation, as well as for ensuring the success of control strategies such as vaccination trials. This is the first review that systematically addresses data available for the diagnosis of TB in wildlife following the Preferred Reporting Items of Systematic Reviews and Meta-Analyses (PRISMA) guidelines. The article also gives an overview of the factors related to host, environment, sampling, and diagnostic techniques which can affect test performance. After three screenings, 124 articles were considered for systematic review. Literature indicates that post-mortem examination and culture are useful methods for disease surveillance, but immunological diagnostic tests based on cellular and humoral immune response detection are gaining importance in wildlife TB diagnosis. Among them, serological tests are especially useful in wildlife because they are relatively inexpensive and easy to perform, facilitate large-scale surveillance and can be used both *ante-* and post-mortem. Currently available studies assessed test performance mostly in cervids, European badgers, wild suids and wild bovids. Research to improve diagnostic tests for wildlife TB diagnosis is still needed in order to reach accurate, rapid and cost-effective diagnostic techniques adequate to a broad range of target species and consistent over space and time to allow proper disease monitoring.

## Introduction

### Overview and importance of wild hosts

Animal tuberculosis (TB) is a globally distributed disease caused by members of the *Mycobacterium tuberculosis* complex (MTC), which can infect humans and a broad range of domestic and wild mammals [1]. TB is a highly relevant zoonosis, causing risk to public health and financial loss due to decreased production, obligatory slaughter of test-positive animals as well as cost of preventive measures. Moreover, it causes threat to conservation strategies in and around protected natural areas [1, 2]. Several wildlife species act as maintenance host, spill over host or host with unknown reservoir status depending on the region. In Europe, Eurasian wild boar (*Sus scrofa*) (Iberian Peninsula), red deer (*Cervus elaphus*) (Iberian Peninsula, Western Austria)*,* fallow deer (*Dama dama*) (Iberian Peninsula) and European badger (*Meles meles*) (British Isles and Atlantic Spain) are regarded as main wildlife MTC reservoir hosts [2–5]. In Africa, wildlife reservoir hosts include common warthog (*Phacochoerus africanus*) (South Africa), African buffalo (*Syncerus caffer*) (South Africa), lechwe antelope (*Kobus leche*) (South Africa) and Eurasian wild boar (North Africa) [6–8]. In addition, wild meerkats (*Suricata suricatta*) (South Africa), African elephant (*Loxodonta africana*) (South Africa), white rhinoceros (*Ceratotheriumsimum*) (South Africa), Nyala (*Tragelaphus angasii*) (South Africa), African lion (*Panthera leo*) (South Africa) and banded mongooses (*Mungos mungo*) (South Africa) are frequently affected with TB [9–13]. In North America, white-tailed deer (*Odocoileus virginianus*) (Michigan, Minnesota, Mexico), wood bison (*Bison bison*) (Canada) and elk (*Cervus canadensis*) (Canada) are the major wildlife hosts identified [14]. In South America, the information with regard to wildlife TB is scarce, even though there are some implications that Brazilian wild boar (*Sus scrofa*) (Brazil) plays a role as disease reservoir [15]. In New Zealand, the Australian brushtail possum (*Trichosurus vulpecula*) acts as primary wildlife reservoir host [16]. In Asia, the disease has been reported in many wild animal species, but there is considerable research gap on this area regarding the role of wildlife in MTC epidemiology. Generally, the above-mentioned species are the potential reservoir hosts in different continents. However, in some regions, disease is on the verge of eradication or infection rate has been considerably reduced due to the intensive diagnosis and prevention protocols [17].

### Relevance of diagnosis in wildlife

Diagnosis in wildlife is (i) a prime step in disease control and management [18], but is also essential (ii) in the evaluation of surveillance strategies, (iii) in pathogenesis, epidemiological and transmission studies as well as (iv) in the assessment of the efficacy of vaccination trials [18–20]. However, diagnosis in wildlife is challenging due to the wide taxonomic diversity, the capture and restraint difficulties inherent to wildlife collection of samples, frequent lack of gold standard diagnostic techniques, lack of knowledge about the true infection status, difficulty in interpretation and conducting experimental studies, as well as limited financial resources [18]. Nevertheless, TB in wildlife is an active area of research.

Many studies have been carried out in order to overcome the problems associated with TB testing and surveillance in wildlife. The lines of investigations include development of new diagnostic techniques like rapid tests [STAT PAK assay or Dual Path Platform (DPP) tests] [10, 21] or the modification of existing ones [21–25] in order to improve the diagnostic efficiency and accuracy in wildlife while remaining practical. Some reviews have addressed TB diagnosis in domestic animals and wildlife, underlining TB diagnostic techniques in the context of disease control and eradication [26–30], identifying some research gaps as well as the need of more reliable approaches for TB diagnosis in wildlife. The present work is the first review that summarizes data available from the currently applicable techniques for TB diagnosis in wildlife, especially highlighting the immunological methods, by means of a systematic system following the Preferred Reporting Items of Systematic Reviews and Meta-Analyses (PRISMA) guidelines. We find that, while culture remains as the gold standard method despite its limited sensitivity (Se), there has been considerable progress in cellular and humoral immunological diagnostic tests for wildlife TB diagnosis. Serological tests are especially useful in wildlife because they are economically attractive, technically easy, enable large-scale surveillance and can be applied both in live or dead animals and, in the latter, in combination with pathology. In farmed wildlife, combinations of cellular and humoral tests could enhance diagnostic accuracy.

## Methods

This study followed the PRISMA recommendations for systematic review reporting [31]. The research question was: which are the studies available for diagnosis of TB in wild mammals, the influence of confounding factors on diagnostic accuracy as well as the attempts made with respect to the improvement in diagnosis?

**Figure 1:**
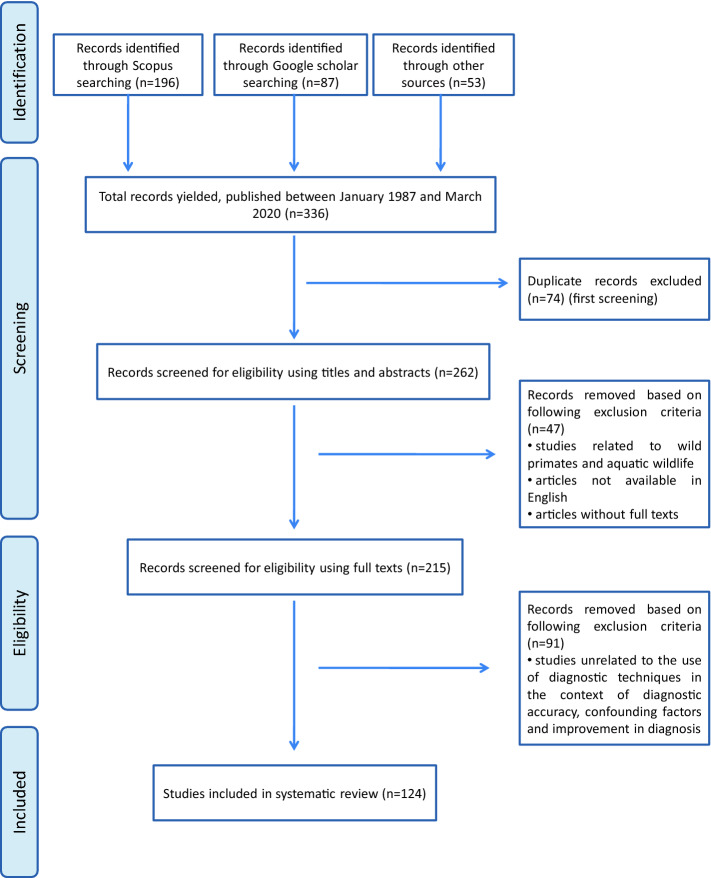
**PRISMA (Preferred Reporting Items for Systematic review and Meta-Analysis) flowchart diagram showing identification and records selection process of studies for the systematic review** **(Adapted from Moher et al. [**31**]).**

Studies were ascertained through a systematic search including four electronic databases (SCOPUS, PubMed, Google Scholar, Google search) available until March 15, 2020. We considered studies in wild mammals in which TB has been reported and we collected data using key elements. Search terms and key elements were combined with the Boolean operators (AND, OR, NOT), resulting in search algorithms which are shown in Additional file 1. The reports obtained for this systematic review were subjected to three screening phases as shown in Figure 1.

## Results and discussion

**Figure 2:**
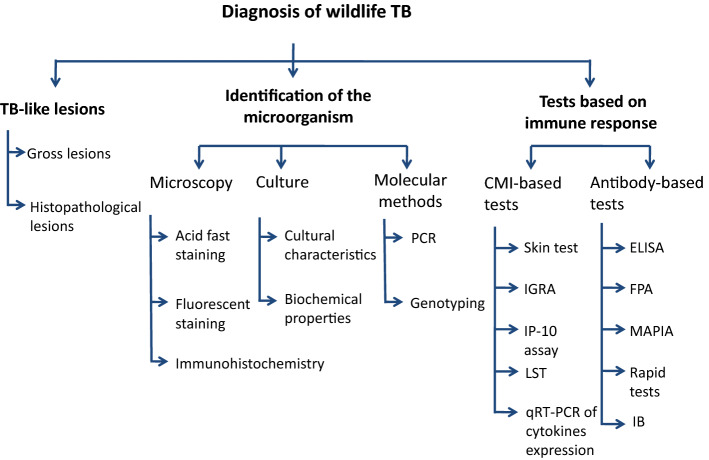
**Principal methods for diagnosis of wildlife tuberculosis.** TB: tuberculosis; CMI: cell-mediated immunity; PCR: polymerase chain reaction; IGRA: interferon gamma release assay; IP-10 assay: interferon gamma-inducible protein 10; LST: lymphocyte stimulation test; qRT-PCR: quantitative reverse-transcription polymerase chain reaction assay; ELISA: enzyme-linked immunosorbent assay; FPA: fluorescence polarization assay; MAPIA: multiantigen print immunoassay; IB: immunoblotting.

A total of 336 articles were retrieved through search engines (196 by Scopus, 87 by Google scholar, 42 by Pubmed and 11 by Google search). Seventy-four articles were removed in first screening and 47 articles were eliminated in second screening. Finally, 124 articles were considered for systematic review after third screening (Figure 1). Different diagnostic tests include detection of TB like lesions (TBL) (macroscopic or microscopic), identification of the microorganism (microscopy/culture and isolation/molecular methods) and immunological methods (cell-mediated immune (CMI) and antibody-based tests). Different tests used for TB diagnosis are schematically represented in Figure 2. The tests can also easily be divided into two groups: tests detecting the pathogen (culture, molecular methods and microscopy) or tests focusing on host response (necropsy, histopathology and immunological methods). Major confounding factors are host, environment, habitat, management factors, prior sensitization, history of vaccination and other infections, sampling factors and technical factors related to diagnosis. The strategies for improving diagnosis comprise selection of appropriate test, proper implementation and interpretation of the test as well as combination of different tests for interpreting the results.

### Principal methods of TB diagnosis

Ideally, a diagnostic test should need a small sample volume with simplicity in its collection and storage before performing the tests and it should be easy to carry out and interpret. It should also be relatively inexpensive, and with high Se and specificity (Sp). An overview of all diagnostic tests is shown in Additional file 2.

#### TB like lesions (TBL)

Presence of TBL is indicative of TB infection and is observed either macroscopically during post-mortem examination or microscopically during histopathological examination.

##### Post-mortem examination

Post-mortem examination is the primary, sensitive and cost-effective method of disease surveillance [32]. This examination can be performed on hunted/dead animals by macroscopic examination of lymph nodes (LNs), and thoracic and abdominal organs, especially lungs to assess the TBL [33]. TBL includes nodular off-white lesions containing caseous material, which may be mineralized in the center and encapsulated by fibrous tissue [23, 34]. The type, severity and place of the lesions can vary depending on the host, route of infection, stage of the disease as well as host–pathogen interaction [35, 36]. Hence, the severity of lesions is negatively associated with protection against disease, which is helpful in the studies related to the efficacy of vaccine experiments [35]. In general, the gross pathological lesions are “noticeable” towards the advanced stages of the disease [23]. However, MTC infection can cause “latent” non-visible lesions in many wild animals, which result in a difficult diagnosis by post-mortem [31, 37]. In Eurasian badger, the majority of infections are latent and hence gross pathological examination provides limited diagnostic Se in this species [34]. Moreover, lesions sometimes are limited to non-classical sites like prescapular or popliteal LNs as a result of MTC transmission by bite wound [38]. The gross TBL cannot be used as a confirmatory TB diagnosis because other non-tuberculous mycobacteria and pathogens other than mycobacteria (i.e. *Corynebacterium pseudotuberculosis*) may cause indistinguishable macroscopic lesions [39]. Hence, post-mortem findings in combination with other diagnostic techniques would be more effective in detecting the disease [40].

##### Histopathological examination

The histological diagnosis involves pathological observation of the “classical tuberculous granuloma”, which consists of a circular lesion formed by cells of inflammatory nature such as macrophages, epithelioid cells, lymphocytes and in some species, Langhans multinucleated giant cells surrounding a central region of developing necrosis (“caseating tubercles”), although non-necrotizing granulomatous encapsulation may also be present [34, 37, 41]. Granulomatous encapsulation with a fibrotic ring and mineralized necrosis in the center is considered as a key factor for containing TB infection [42]. Histopathological examination is not 100% specific for detecting MTC infection due to the detection of similar lesions in other non-tuberculous mycobacteria infections, therefore it requires complementary techniques for confirmation [43]. Despite of that it has many advantages; among them are that focal/latent lesions can be detected as well as different histopathological studies have helped to discern target organs for sampling, i.e. tonsils and submandibular LNs in wild boar, mesenteric LNs in red deer or hepatic LNs in badgers [34, 37].

#### Identification of the microorganism

Identification of MTC organisms is a confirmatory method in TB diagnosis that can be performed by direct microscopy, culture or molecular methods for identification.

##### Microscopy

Ziehl–Neelsen (ZN) staining is used to identify acid-fast bacteria, mainly mycobacteria, and can increase the reliability of TB diagnosis [44]. This is a simple, rapid and economical method for the detection of mycobacteria, but it is not 100% specific due to the detection of other non-tuberculous mycobacteria [42]. Samples can be collected from a slaughtered/hunted animal or a live animal (samples of tracheobronchial washes). ZN staining is commonly used in histological sections which improves the diagnostic Se.

Immunofluorescence and immunohistochemistry include the staining of mycobacteria using monoclonal or polyclonal antibodies. These techniques provide more accurate results than acid fast staining [43], but they are laborious and less economical, along with the fact that sometimes polyclonal antibodies can give non-specific results due to cross-reactions with other non-tuberculous mycobacteria [45].

##### Microbiological culture

Culture of microorganism and identification are considered as the gold standard method for the diagnosis of TB in all wild animals [46]. It is employed as a post-mortem diagnostic method by collecting the samples especially from organs with TBL [19, 36] or pooled LN samples in case of non-visible TBL [28]. Culture from bronchoalveolar lavage (BAL)/tracheal washing can be used for ante-mortem diagnosis in some wild animals like lion [13], badger [43] and wild meerkat [9]. However, Se can be variable due to the lack of active shedding of the microorganism from infected animals and hence the absence of mycobacteria in the collected sample [47]. In addition, the decontamination step prior to MTC culture can adversely affect the viability of mycobacteria, especially when the number of viable microorganisms is low, and it may, therefore, lead to false negative results in the culture [24]. In elephants, trunk wash culture is the officially recommended ante-mortem diagnostic test in USA [10], but the contamination by other pathogens is a major problem [48]. The Se of culture and isolation varies depending on the stage of the disease (latent or noticeable), the number and selection of tissues processed and on the sample quality [49]. Culture is expensive, time-consuming and requires biosafety level 3 laboratories [49]. However, live sampling is highly relevant, as it is important to detect the excretion of pathogens and thereby determine the possibility of transmission (continuous studies are possible with re-sampling). It is also important for determining the infection rate before and after applying control methods like vaccination as well as to detect the safety of vaccine [19, 30]. Identification of the microorganism after cultural isolation can be done by colony characteristics, biochemical tests or nucleic acid recognition methods [50]. Moreover, culture is performed on specific medium which supports the confirmation that the bacteria is from the MTC.

##### Molecular identification

Nucleic acid recognition methods can be applied using DNA extracted directly from tissue or clinical samples (not blood) or from the growing colonies. DNA extraction from growing colonies is more efficient since growing colonies usually contain higher bacterial load (approximately more than 50 colony forming units (CFU)/g) and are also usually less contaminated than tissues or clinical samples, which facilitates the extraction. The diagnosis of MTC by direct polymerase chain reaction (PCR) is fast and highly sensitive, showing great value in epidemiological studies [51] and, it is highly useful to reach immediate treatment decisions in some species [48]. The test has variable Se due to absence of organism in collected sample, which in turn depends on many factors like stage of disease, intensity of infection etc. Sample quality is a major factor regarding test result because presence of DNAases can degrade DNA resulting in false negative result. Moreover PCR requires costly reagents and equipment [48, 51].

Another nucleic acid recognition method is genotyping, which comprises spoligotyping, deep sequencing, Restriction Fragment Length Polymorphism (RFLP) and Mycobacterial Interspersed Repetitive Units-Variable Number of Tandem Repeats (MIRU-VNTR) analysis [52]. Genotyping can also be used to discriminate different members and strains of MTC species, to elucidate transmission patterns, to perform large-scale molecular epidemiological studies [53] or to detect outbreaks and their sources by MIRU-VNTR [54].

#### Tests based on immune response

The immunological TB diagnosis is based on CMI and humoral mediated diagnostic tests using different antigens. The main antigen used in diagnostics is bovine tuberculin or bovine purified protein derivative (bPPD), which is a combination of proteins extracted from *M. bovis*. Another antigen used in diagnostics is lipoarabinomannan (LAM), which is a glycolipid as well as a virulence factor associated with MTC [55]. The specific antigens used in TB diagnosis include proteins from *M. bovis* MPB83 and MPB70, which are homologous proteins within MTC members, but have difference in electrophoretic mobility and isoelectric point [56], early secretory antigenic target-6 kDa (ESAT-6) and culture filtrate protein-10 kDa (CFP-10), which are virulence proteins of MTC absent in most of the non-tuberculous mycobacteria and *M. bovis* Bacillus Calmette Guerin (BCG) [57], and cell wall proteins like Rv3615c and Rv3020c, absent in most of the non-tuberculous mycobacteria and *M. bovis* BCG [58]. The antigen (Ag) 85A is a secretory protein of *M. tuberculosis* and BCG, but it does not have much relevance in diagnosis of animal TB [20]. The P22 antigen is a recently immunopurified protein complex from bPPD comprising mainly the proteins MPB70, MPB83, ESAT-6 and CFP-10 [59]. The details in immunological techniques used for TB diagnosis of wildlife are listed in Tables 1, 2, 3, 4, 5, 6, 7 and 8.

**Table 1:** **Details of immunological diagnosis in Cervidae.**

Assay test	Species	N/E	n_Se_ + n_Sp_	Antigens	Se (%)	Sp (%)	References
Skin test (SCITT)	Red deer	N + E	60 + 1157	bPPD, aPPD	91.4	98.7	[144]
	Red deer	N	218	bPPD, aPPD	NE	46.9	[126]
	Elk	N	7 + 3	bPPD, aPPD	100	100	[145]
	Reindeer	E	13 + 4	bPPD, aPPD	92	25	[146]
	White tailed deer	N + E	60 + 56	bPPD, aPPD	97	81	[61]
	Fallow deer	N	21	bPPD, aPPD	80.1	NE	[21]
IGRA	White tailed deer	E	91 + 44	bPPD, aPPD	74	98	[83]
	Elk	N	51	bPPD, aPPD	NE	90^a^, 78^b^	[79]
				ESAT-6/CFP-10	NE	100^c^ 96^d^	
	Reindeer	N	106	bPPD, aPPD	NE	91^a^ 83^b^	
				ESAT-6/CFP-10	NE	94^c^, 87.5^d^	
	White tailed deer	N	95	bPPD	NE	98^a^, 92^b^	
				ESAT-6/CFP-10	NE	97^c^, 95^d^	
	Red deer	E	15	bPPD	92.8^e^, 100^f^, 75^g^	100	[23]
				P22	92.8^e^, 100^f^, 87.5^g^	100	
				ESAT-6/CFP-10	92.3^e^, 100^f^, 75^g^	100	
				Rv3615c	40^e^, 26.6^f^, 37.5^g^	100	
				Rv3020c	66.6^e^, 100^f^, 87.5^g^	100	
LST	Elk	N	66 + 324	bPPD	70	74	[147]
	Elk	N	33 + 450	bPPD	83	64	[104]
	Red deer	N	39 + 16	bPPD	95	44	[68]
				MPB70	72	50	
	Red deer-elk hybrid	E	10 + 15	bPPD	65.7	92.5	[84]
qRT-PCR of cytokines expression	Red deer-elk hybrid	E	10 + 15	bPPD	78.6	97.5	[84]
ELISA	Red deer	N	104 + 56	bPPD MPB70	88 80	52 79	[68]
	Red deer	N	94 + 217	bPPD, MPB70, aPPD	45.7	100	[126]
	Red deer	E	15 + 15	Ethanol Extract of *M. bovis*	86.7	93.3	[102]
	Red deer	N	221 + 204	bPPD P22	70.1 70.1	91.6 99	[24]
	Elk	N	108 + 48	MPB83	49.1	97.9	[138]
	Fallowdeer	N	73 + 157	bPPD	51	96	[120]
	White tailed deer	N	12 + 329	LAM enriched antigen	66.7^h^, 58.3^i^	95.1^h^ 97.3^i^	[113]
	Reindeer	E	11 + 4	LAM	100	50	[55]
BTB	Red deer	N	87 + 200	bPPD, aPPD	90.8	98	[126]
FPA	Red deer and elk	N	16	MPB70	81	NE	[91]
	Elk	N	33 + 450	MPB70	40	81	[104]
	Red deer-elk hybrid	E	10	MPB70	33.33	NE	[75]
RT	White tailed deer	N/E	28 + 435	ESAT-6, CFP-10, MPB83	75	98.9	[109]
Cervid TB STAT-PAK	Elk	N	31 + 842	ESAT-6, CFP-10, MPB83	87.1	98.3	[148]
	Elk	N	33 + 450	ESAT-6, CFP-10, MPB83	62	87	[104]
	Elk Fallow deer	N	34 + 141 32 + 107	ESAT-6, CFP-10, MPB83	82 91	93 91	[107]
	Fallow deer	N	21	ESAT-6, CFP-10, MPB83	80.1	NE	[21]
	Red deer-elk hybrid	E	10	ESAT-6, CFP-10, MPB83	72.5	NE	[75]
	White tailed deer	N	22 + 724	ESAT-6, CFP-10, MPB83	54.5	98.1	[113]
	Red deer	N/E	52 + 105	ESAT-6, CFP-10, MPB83	86.5	83.8	[119]
	Mixed Deer sp.	N	7 + 425	ESAT-6, CFP-10, MPB83	85.7^j^	94.8^j^	[119]
DPP Vet TB	Elk Fallow deer	N	34 + 141 32 + 107	ESAT-6, CFP-10, MPB83	79 91	98 99	[107]
	Fallow deer	N	73 + 157	ESAT-6, CFP-10, MPB83	71	88	[120]
	Red deer	N/E	52 + 105	ESAT-6, CFP-10, MPB83	84.6	91.4	[119]
	White tailed deer	N/E	63 + 903	ESAT-6, CFP-10, MPB83	65.1	97.8	[121]
MAPIA	Elk Fallow deer	N	34 32	*M. bovis* antigens^k^	82 97	NE NE	[107]
	Red deer-elk hybrid	E	10	*M. bovis* antigens^k^	76.7	NE	[75]
	White tailed deer	N	22 + 727	*M. bovis* antigens^k^	68.2	97.1	[113]
	Reindeer	E	11 + 23	*M. bovis* antigens^k^	100	85	[55]
IB	White tailed deer	N	20 + 671 13 + 333	MPB83 Whole cell sonicate	55 46.2	99.3 92.5	[113]
	Reindeer	E	11 + 4	Whole cell sonicate	90.9	50	[55]

N/E: natural or experimental infection; n_Se_: number of TB positive animals used for evaluation of Se; n_Sp_: number of TB negative animals used for evaluation of Sp; Se: sensitivity; Sp: specificity; SCITT: single comparative intradermal tuberculin skin test; LST: lymphocyte stimulation test; qRT-PCR: quantitative reverse-transcription polymerase chain reaction assay; IGRA: interferon gamma release assay; ELISA: enzyme linked immunosorbent assay; BTB: blood tuberculosis test*;* FPA: fluorescent polarization assay; MAPIA: multiantigenimmunoprint assay; Cervid TB STAT-PAK and DPP Vet TB: lateral flow tests (Chembio Diagnostic Systems, Inc., USA); RT: lateral flow rapid test (name not mentioned-Chembio Diagnostic Systems, Inc., USA); NE: not estimated; IB: immunoblotting

^a^ Cut off: OD of bPPD–aPPD and bPPD–PBS > 0.1.

^b^ Cut off: OD of bPPD–aPPD and bPPD–PBS > 0.05.

^c^ Cut off: OD of ESAT-6:CFP-10–PBS > 0.1.

^d^ Cut off: OD of ESAT-6:CFP10–PBS > 0.05.

^e^ Se of experimentally infected animals at 15 days post-infection (dpi).

^f^ Se of experimentally infected animals at 30 dpi.

^g^ Se of experimentally infected animals at 60 dpi (cut off for e, f, g: for bPPD and P22, OD of bPPD–aPPD and bPPD–PBS > 0.05, for specific antigens like ESAT-6:CFP10, Rv3615c and Rv3020c, OD of specific antigen–PBS > 0.05).

^h^ Cut off: OD ≥ 0.25.

^i^ Cut off: OD ≥ 0.3.

^j^ Se and Sp were evaluated based on mixed species of deer consisting of fallow deer, roe deer and red deer.

^k^ ESAT-6, CFP-10, MPB59, MPB64, MPB70, MPB83, 16-kDa protein, 38-kDa protein, CFP-10/ESAT-6 and 16 kDa protein/MPB83, and two native antigens, bovine PPD and *M. bovis* culture filtrate.

**Table 2:** **Details of immunological diagnosis in wild suids.**

Assay test	Species	N/E	n_Se_ + n_Sp_	Antigen	Se (%)	Sp (%)	References
Skin test (SCITT)	Eurasian wild boar	E	4 + 31	bPPD, aPPD	77-100^a^	48.4–77.4^a^	[22]
	Common warthog	N	16 + 18	bPPD, aPPD	81	100	[8]
Skin test (SIT)	Common warthog	N	16 + 18	bPPD, aPPD	69	100	
ELISA- protein G	Eurasian wild boar	N	96 + 104	bPPD	79.2	100	[133]
ELISA- IgG	Eurasian wild boar, domestic pig	N	277 + 366	bPPD P22	77.3 84.1	97.3 98.4	[25]
	Eurasian wild boar	N E	30 + 25 51 + 9	P22 P22	96.7 84.3	100 100	[125]
ELISA- protein A&G	Eurasian wild boar	N	22 + 43	MPB83	86	100	[149]
	Common warthog	N	16 + 19	bPPD	88	89	[97]
	Common warthog	N	25	bPPD	92	NE	[150]
ELISA TB-VK	Eurasian wild boar	N	73 + 112	bPPD	72.6	96.4	[137]
	Common warthog	N	16 + 19	bPPD	88	79	[97]
	Common warthog	N	25	bPPD	86	NE	[150]
ELISA-INgezim TB porcine	Eurasian wild boar	N E	30 + 25 51 + 9	MPB70, MPB83	100 92.1	100 100	[126]
INgezim Tuberculosis DR	Eurasian wild boar	N E	30 + 25 51 + 9	MPB83	93.3 86.2	100 100	[125]
RT	Eurasian wild boar	N	64 + 113	ESAT-6, CFP-10, MPB83	76.6	97.3	[109]
DPP VetTB	Warthog Eurasian wild boar	N	23 + 35 56 + 30	ESAT-6, CFP-10, MPB83	82.6 80.4	91.4 96.7	[110]
	Eurasian wild boar	N	96 + 104	ESAT-6, CFP-10, MPB83	89.6	90.4	[133]
	Common warthog	N	16 + 19	ESAT-6, CFP-10, MPB83	75	89	[97]
INgezim TB-CROM	Eurasian wild boar	N E	30 + 25 51 + 9	MPB83	93.3 90.2	96 100	[125]

N/E: natural or experimental infection; Se: sensitivity; Sp: specificity; n_Se_: number of TB positive animals used for evaluation of Se; n_Sp_: number of TB negative animals used for evaluation of Sp; ^a^ range of values due to the difference in reading criteria; SIT: single intradermal tuberculin test; SCITT: single comparative intradermal tuberculin skin test; IGRA: interferon gamma assay; ELISA: enzyme linked immunosorbent assay; ELISA TB-VK: Commercial ELISA kit (Vacunek, Spain); ELISA- INgezim TB porcine and INgezim Tuberculosis DR: Commercial ELISA kit (INGENASA, Spain); RT: lateral flow rapid test (name not mentioned-Chembio Diagnostic Systems, Inc., USA); DPP VetTB: rapid lateral flow kit (Chembio Diagnostic Systems, Inc., USA), INgezim TB-CROM: rapid lateral flow kit (INGENASA S.L., Spain).

**Table 3:** **Details of immunological diagnosis in European badger.**

Assay test	N/E	n_Se +_n_Sp_	Antigen	Se (%)	Sp (%)	References
Skin test	N	10 + 37	bPPD, NT, whole killed bovine tubercle bacilli	70	73	[64]
IGRA	N	46 + 185	bPPD, aPPD	74.5 80.9^a^	93.6 93.6^a^	[80]
			ESAT-6/CFP-10	60.9 60.9 ^a^	94.6 94.6 ^a^	
	N	ND	bPPD, aPPD	79.9	95	[143]
	N	39 + 147	bPPD, aPPD	84.6^b^	92.5^b^	[96]
		7 + 40		57.1^c^	97.5^c^	
	N	ND	bPPD, aPPD	52	97	[142]
LTA	N	8 + 13	bPPD, aPPD	87.5	84.6	[92]
qRT-PCR of cytokines expression	N	53 + 194	bPPD, ESAT-6/CFP-10	64.2	93.3	[93]
ELISA	N	25 + 28	P22	80^d^, 76^e^	85.7^d^, 85.7^e^	[99]
	E	34 + 36		82^d^,79^e^	80.56^d^, 83.3^e^	
Brock test	N	8 + 13	MPB83	62.5	100	[92]
	N	41 + 33	MPB83	46	82	[95]
	N	340 + 817	MPB83	54.7^b^	93^b^	[96]
		39 + 238		53.9^c^	96.6^c^	
	N	51 + 193	MPB83	52.9	90.7	[93]
	N	78 + 100	MPB83	47.4	89	[101]
Dachs TB ELISA	N	41 + 33	MPB83	61	82	[95]
MAPIA	N	78 + 100	*M. bovis* antigens^f^ *M. bovis* antigens^g^	48.7 59	88 84	[101]
BrockTB STAT-PAK	N	340 + 817 39 + 238	ESAT-6, CFP-10, MPB83	49.7^b^ 56.4^c^	92.5^b^ 96.2^c^	[96]
	N	ND	ESAT-6, CFP-10, MPB83	50.4	96.9	[143]
	N	NM	ESAT-6, CFP-10, MPB83	59.09	66.67	[139]
	N	ND	ESAT-6, CFP-10, MPB83	58	97	[142]
DPP Vet TB	N	38 + 418	ESAT-6, CFP-10, MPB83	50	95	[123]
RT	N	454 + 1078	ESAT-6, CFP-10, MPB83	50.7	93.1	[109]
	N	78 + 100	MPB83, TBF 10	52.6	95	[101]

N/E: natural or experimental infection; n_Se_: number of TB positive animals used for evaluation of Se; n_Sp_: number of TB negative animals used for evaluation of Sp; Se: sensitivity; Sp: specificity; IGRA: interferon gamma release assay; ELISA: enzyme linked immunosorbent assay; LTA: comparative lymphocyte transformation assay; qRT-PCR: quantitative reverse-transcription polymerase chain reaction assay; MAPIA: multiantigenimmunoprint assay; NT: new tuberculin; Brock TB STAT-PAK: lateral flow rapid test (Chembio Diagnostic Systems, Inc., USA); RT: rapid test (name not mentioned).

^a^Se and Sp based on rabbit monoclonal antiserum (mEIA) instead of polyclonal pair (pEIA) of antibodies in other studies; ND: not defined, Se and Sp are calculated based on Bayesian analysis; NM: not mentioned in the article; NE: not estimated.

^b^Se or Sp in adults.

^c^Se or Sp in cubs.

^d^Indirect ELISA.

^e^Competitive ELISA.

^f^Recombinant antigens only i.e., ESAT-6, CFP-10,MPB59, MPB64, MPB70, MPB83, 16-kDa protein, 38-kDa protein, CFP-10/ESAT-6 and 16 kDa protein/MPB83.

^g^Recombinant antigens and *M. bovis* culture filtrate.

**Table 4:** **Details of immunological diagnosis in wild bovids.**

Assay test	Species	N/E	n_Se_ + n_Sp_	Antigens	Se (%)	Sp (%)	References
Skin test (CFT)	Wild bison	N	ND	bPPD	57.6	80.3	[105]
	Wild bison	N	2 + 24	bPPD	50	88	[111]
Skin test (SCITT)	African buffalo	N	51	bPPD, aPPD	86.3	NE	[88]
IGRA	African buffalo	N + E	149 + 344	bPPD, aPPD	92.1	68.3	[81]
	African buffalo	N	8	bPPD, aPPD	100	NE	[7]
				PC-HP, PC-EC	75	NE	
	African buffalo	N	44	ESAT-6, CFP-10, TB-7.7	71	NE	[86]
				PC-EC	91	NE	
				PC-HP	95	NE	
	African buffalo	N	51 + 70	ESAT-6, CFP-10, TB-7.7	80.4	100	[88]
IP-10 assay	African buffalo	N	44 + 40	PC-HP	93.2	90	[86]
				PC-EC	81.8	92.5	
				ESAT-6, CFP-10, TB-7.7	86.4	92.5	
	African buffalo	N	32 + 39	ESAT-6, CFP-10, TB-7.7	94	92	[87]
	African buffalo	N	51 + 70	ESAT-6, CFP-10, TB-7.7	88.2	100	[88]
MAPIA	Wild bison	N	12 + 70	*M. bovis* antigens^a^	92	97	[111]
FPA	Wild bison	N	ND	MPB70	4.4	83.2	[105]
	Wild bison	N	9 + 57	MPB70	67	34	[111]
Idexx ELISA	African buffalo	N	8	bPPD	37.5	NE	[7]
BovidTB STAT-PAK	African buffalo	N	100 + 100	ESAT-6, CFP-10, MPB83	33	90	[114]
VetTB STAT-PAK	Wild bison	N	ND	ESAT-6, CFP-10, MPB83	12.7	98.4	[105]
RT^1^	African buffalo	N	100 + 100	NM	23	94	[114]
RT^2^	Wild bison	N	12 + 70	ESAT-6, CFP-10, MPB83	67	99	[111]

N/E: natural or experimental infection; n_Se_: number of TB positive animals used for evaluation of Se; n_Sp_: number of TB negative animals used for evaluation of Sp; Se: sensitivity; Sp: specificity; CFT: caudal fold test; SCITT: single comparative intradermal tuberculin skin test; IGRA: interferon gamma release assay; IP-10: interferon gamma-inducible protein 10; PC-EC: contains ESAT-6- and CFP-10-derived peptides; PC-HP contains ESAT-6- and CFP-10-derived peptides and 4 mycobacterial antigens (including Rv3615); MAPIA: multiantigenimmunoprint assay; FPA: fluorescent polarization assay; ELISA: enzyme linked immunosorbent assay; Idexx ELISA: commercial ELISA (Idexx Laboratories, Inc., Westbrook, ME, USA); Bovid TB STAT-PAK and DPP Vet TB: lateral flow tests (Chembio Diagnostic Systems, Inc., USA); RT^1^: rapid test (Anigen, Animal Genetics, Inc., South Korea-name of the test not mentioned); RT^2^: lateral flow rapid test (Chembio Diagnostic Systems, Inc., USA); NE: not estimated; NM: not mentioned; ND: not defined, Se and Sp estimated based on Bayesian analysis (absence of gold standard).

^a^ ESAT-6, CFP-10, MPB59, MPB64, MPB70, MPB83, the16-kDa protein, the 38-kDa protein, two fusion proteins comprising CFP-10/ESAT-6 and the 16 kDa protein/MPB83, and two native antigens, bovine PPD and *M. bovis* culture filtrate.

**Table 5:** **Details of immunological diagnosis in elephants.**

Assay test	Species	N/E	n_Se_ + n_Sp_	Antigens	Se (%)	Sp (%)	References
Skin test^b^	Asian elephant	N	6 + 31	bPPD alone or with aPPD	16.7	74.2	[65]
Elephant TB Stat-Pak	African/Asian elephant	N	26 + 147	ESAT-6, CFP-10, MPB83	100	95.2	[10]
	African/Asian elephant	N	14	ESAT-6, CFP-10, MPB83	100	NE	[122]
MAPIA	African/Asian elephant	N	26 + 147	*M. bovis* antigens^c^	100	100	[13]
DPP VetTB assay	African/Asian elephant	N	26 + 147	ESAT-6, CFP-10, MPB83	100	100	[10]
	African/Asian elephant	N	14	ESAT-6, CFP-10, MPB83	100	NE	[122]
ELISA	African/Asian elephant	N	7 + 40	CF	100	95	[98]
				bPPD	85.7	97.5	
				MPB70	28.6	97.5	
				ERD	71.4	85	
				RA	57.1	90	
	Asian elephant	N	8 + 41	RA, CF, bPPD MPB70, ERD, aPPD	100^a^	87.8^a^	[65]
			3 + 25	CF, LAM, aPPD	33.3^a^	100^a^	
			8 + 55	*N*-lauryl-sarcosyl extract of *M. bovis*, bPPD, aPPD, and *M. intracellulare* PPD	87.5^a^	83.6^a^	
BTB	Asian elephant	N	6 + 31	bPPD, aPPD	83.3	51.6	[65]

N/E: natural or experimental infection; n_Se_: number of TB positive animals used for evaluation of Se; n_Sp_: number of TB negative animals used for evaluation of Sp; Se: sensitivity; Sp: specificity; ELISA: enzyme linked immunosorbent assay; Elephant TB Stat-Pak and DPP VetTB assay: lateral flow rapid tests **(**Chembio Diagnostic Systems, Inc., USA); MAPIA: multiantigenimmunoprint assay; CF: *M. bovis* AN5 culture filtrate; ERD: lipoarabinomannan antigen Erdman strain of *M. tuberculosis*; RA: lipoarabinomannan antigen H37 Ra strain of *M. tuberculosis*; LAM: lipoarabinomannans; BTB: blood tuberculosis test.

^a^ Se and Sp are determined excluding the weak responses.

^b^ Single intradermal tuberculin test, caudal fold test as well as single comparative intradermal tuberculin skin test depending on the herd.

^c^ ESAT-6, CFP-10, MPB59, MPB64, MPB70, MPB83, the16-kDa protein, the 38-kDa protein, CFP-10/ESAT-6 and the 16 kDa protein/MPB83, bovine PPD and *M. bovis* culture filtrate.

**Table 6:** **Details of immunological diagnosis in African lion.**

Assay test	N/E	n_Se_ + n_Sp_	Antigens	Se (%)	Sp (%)	References
SIT	N	52 + 32 44	bPPD	86.5 NE	81.2 100^a^	[62]
SCITT	N	52 + 32 44	bPPD, aPPD	80.8 NE	81.2 100^a^	[62]
	N	8	bPPD, aPPD	63	NE	[115]
Elephant TB Stat-Pak	N	11	ESAT-6, CFP-10, MPB83	64	NE	[115]
DPP Vet TB	N	10	ESAT-6, CFP-10, MPB83	70	NE	[115]

N/E: natural or experimental infection; n_Se_: number of TB positive animals used for evaluation of Se; n_Sp_: number of TB negative animals used for evaluation of Sp; Se: sensitivity; Sp: specificity; SIT: single intradermal cervical test; SCITT: single comparative intradermal tuberculin skin test: NE: not estimated; Elephant TB Stat-Pak and DPP Vet TB are lateral flow rapid tests (Chembio Diagnostic Systems, Inc., USA).

^a^ Sp of the test when 44 samples from uninfected population are considered and only 11 of which are tested negative by culture.

**Table 7:** **Details of immunological diagnosis in possum.**

Assay test	N/E	n_Se_ + n_Sp_	Antigens	Se (%)	Sp (%)	References
ELISA	N	29 + 100	*M. bovis* culture filtrate MBP70	45^a^ 21^a^ 28^b^	96^a^ 98^a^ 99^b^	[100]
RT	N	38 + 91	ESAT-6, CFP-10, MPB83	44.7	85.7	[109]

N/E: natural or experimental infection; n_Se_: number of TB positive animals used for evaluation of Se; n_Sp_: number of TB negative animals used for evaluation of Sp; Se: sensitivity; Sp: specificity; ELISA: enzyme linked immunosorbent assay; RT: lateral flow rapid test (name not mentioned- Chembio Diagnostic Systems, Inc., USA).

^a^ Se or Sp of ELISA where blocking antibody against MBP70 is not used.

^b^ Se or Sp of blocking ELISA where a monoclonal antibody against MPB70 is used.

**Table 8:** **Details of immunological diagnosis in wild meerkat.**

Assay test	N/E	n_Se_ + n_Sp_	Antigens	Se (%)	Sp (%)	References
MAPIA	N	ND	*M. bovis* antigens^*^	90	48	[9]
BovidTB STAT-PAK	N	ND	ESAT-6, CFP-10, MPB83	43	85	[9]

MAPIA: multiantigenimmunoprint assay; BovidTB STAT-PAK: lateral flow test (Chembio Diagnostic Systems, Inc., USA); N/E: natural or experimental infection; n_Se_: number of TB positive animals used for evaluation of Se; n_Sp_: number of TB negative animals used for evaluation of Sp; Se: sensitivity; Sp: specificity; ND: not defined, Se and Sp were estimated based on Bayesian analysis (absence of gold standard).

^a^ESAT-6, CFP-10, MPB59, MPB64, MPB70, MPB83, the16-kDa protein, the 38-kDa protein, CFP-10/ESAT-6 and the 16 kDa protein/MPB83, bovine PPD and *M. bovis* culture filtrate.

##### CMI based diagnostics

Cell mediated immunity based diagnostic tests are based on the type IV or delayed hypersensitivity in which sensitized T cells produce cytokines, mainly interferon gamma (IFNγ), interleukin-2 (IL-2) and IL-16, as well as chemokines resulting in mast cell degranulation and production of histamine [60]. They are mainly used to detect initial stages of the disease.

###### Skin test

Skin test is an ante-mortem TB diagnostic test that is not usually applicable to wildlife due to the need to handle animals twice over a 2–3 day interval [61]; however it can be used in some captive wildlife and zoo animals. Generally, the test involves the intradermal inoculation of tuberculous antigen (mostly bPPD) and measuring the skin thickness after 72 h, which is called single intradermal tuberculin skin test (SIT). Another type of skin test is single comparative intradermal tuberculin skin test (SCITT) in which avian purified protein derivative (aPPD) is also used in addition to bPPD in order to avoid non-specific reaction of *Mycobacterium avium* complex (MAC) [22]. The optimum time for second reading of skin test is 72 h [21]. Skin testing is relatively less expensive, but it does not support differentiation of infected and vaccinated animals (DIVA) testing. Skin test has been tested in different wild animal species with varying level of diagnostic accuracy. Sites of inoculation also differ depending on the species assayed, like neck region in deer [21], lion [62], nyala [12] and African buffalo [63]; chest wall over the posterior rib cage in badger [64]; caudal fold (caudal fold test-CFT) in elephant [65] and African buffalo [66]; inguinal region in wild boar [22]; or caudal part of the ear in warthog and pygmy hippopotamus [8, 67]. In cattle, the interpretation of SIT is based on the scoring of reactions higher than 4 mm as *standard reactor*, higher than 2 mm and less than 4 mm as *inconclusive* reactor and less than 2 mm as *negative* reactor. In SCITT, any animal with a skin fold increase greater than 2 mm to bPPD and bPPD > aPPD (at least 1 mm) is considered as TB reactor [13, 68].

This method is the official ante-mortem test in farmed deer as approved by Office international des epizooties (OIE) [69]. In wild bovid species like African buffalo or Kafue lechwe, skin test is routinely used for diagnosis of TB as well as in test and slaughter programs [70, 71]. Even so, in many other species, this technique is less reliable or has less diagnostic accuracy or rather limited information is available. Black skin of many species of swine restricts the implementation of skin test [72]. The test is not practical in pachyderm animals like elephant and rhinoceros [73]. In possum and badger, the test is not reliable due to the weak responses produced [74]. Generally, tuberculin skin test has some drawbacks including the need to handle the animals twice in a 72-h period and stress associated with this double handling which can influence the results [75], technical variability [76], low Sp [77] and reduced Se towards later stages of the disease [78]. Moreover, the possibility of acute stress in tested animals could induce high levels of cortisol, resulting in reduced IFNγ response [66, 79].

###### Whole blood interferon gamma release assay (IGRA)

The IGRA is used as an alternative or supplementary assay to the skin test. The test involves the measurement of CMI response in vitro by an assay that detects the IFNγ produced by peripheral blood mononuclear cells (PBMCs) exposed to bPPD (or specific antigens like ESAT-6/CFP-10, P22, Rv3615c or Rv3020c) and aPPD antigens. The assay has been tested in deer [23], African buffalo [63], badger [80], elephant [73], white rhinoceros [11] and wild boar [36]. Briefly, the assay consists of two stages. First, heparinized whole blood is incubated with antigens (i.e., PPDs, specific MTC antigens along with positive controls as mitogens or superantigens (pokeweed mitogen, phytohemagluttinin or staphylococcal enterotoxin B) and negative controls as phosphate buffered saline (PBS) or Roswell Park Memorial Institute (RPMI) Medium) for 18–24 h to induce production and release of IFNγ predominantly by T lymphocytes. Secondly, IFNγ present in the plasma supernatants is quantified in a sandwich ELISA using two species specific antibodies against IFNγ (capture and detection antibody) [23, 63, 80]. Usually, the difference in the optical density (OD) of antigens with negative control and aPPD is taken into consideration for interpreting the results. The interpretation of the test varies with many factors like species, type of antigen/antibody used, preferred Se/Sp, as well as purpose of the test [23, 81].

IGRA has many practical advantages over the skin test in wildlife, as the test avoids the stimulation of live animal with mycobacterial antigens, as well as it needs only a single sample collection and less technical variability compared to skin test [23, 81]. Moreover, IGRA can be applied sequentially to get a conclusive result if there is a doubt or inconclusive result in the skin test, as well as in association with other antibody mediated diagnostic measures to improve the Se and Sp [23]. However, the test involves logistical constraints such as strict laboratory conditions and the need for fast processing of samples [82].

In wild bovids, like African buffalo and nyala, the commercial IGRA test usually used is Bovigam assay with bPPD (Prionics, Zurich, Switzerland) [12]. Cervigam assay (Pfizer Animal Health, New York, USA) was the commercial IGRA test developed for use in deer [83, 84]. However, Cervigam assay is not commercially available nowadays, since a lack of adequate response to mycobacterial antigens has been reported in infected white-tailed deer, elk and fallow deer [79]. Offering the greatest potential for the improvement of Sp, the use of specific antigens like ESAT-6, CFP-10, P22, TB-7, Rv3615c or Rv3020c in IGRA has been carried out in many wild animal species like African buffalo [63], badger [80] and red deer [23]. In African buffalo, the commercially available Bovigam PC-EC assay, based on ESAT-6:CFP-10 proteins, and Bovigam PC-HP assay, based on ESAT-6/CFP-10 proteins, peptides simulating Rv3615c and three additional mycobacterial antigens, are promising diagnostic approaches [85–87]. In red deer, IGRA with antigens bPPD, P22 and the combination of ESAT-6/CFP-10 and Rv3020c was able to detect the infection as early as 15 days post-experimental challenge [23]. Moreover, IGRA based on ESAT-6/CFP-10 is able to differentiate between BCG vaccinated and infected animals [20, 23, 36]. On the other hand, the use of specific or purified antigens can result in lack of suitable Se in badger [80]. Nevertheless, there are some strategies focusing on new antigen stimulating platforms in order to counteract this loss of Se. Thus, modified QuantiFERON^®^ TB Gold In-Tube (mQFT) system is an IGRA which utilizes peptides simulating ESAT-6 and CFP-10 antigens, as well as TB7 in three blood collection tubes for the stimulation of PBMCs to produce IFNγ, and it provided better Sp without losing Se in African buffalo [63, 71, 88], warthog [89] and white rhinoceros [11].

###### IGRA enzyme-linked immunospot assay (ELISPOT)

A direct ELISPOT assay has also been conducted in some studies in badgers with a high diagnostic value [20, 52]. The assay is usually based on net bPPD response and the use of the other antigens only aims to increase Sp. The assay detects IFNγ produced by PBMCs stimulated with bPPD and aPPD, CFP-10/ESAT-6, Ag85, P22 or mitogen Concanavalin A as positive control, all diluted in RPMI complete medium for 16–20 h in wells pre-coated with monoclonal antibody against IFNγ. The IFNγ producing cells are detected with biotinylated monoclonal antibody and the ELISPOT results are expressed as number of spot forming units/million cells. The main limitations of this technique are economical and technical as the samples must be collected in live anesthetized badgers and must be processed immediately.

###### IFNγ-inducible protein 10 (IP-10) assay

IFNγ-inducible protein 10 (IP-10) is a chemokine induced by IFNγ which plays a role in type IV hypersensitivity reactions. The assay protocol is the same as that of IGRA, involving the stimulation of whole blood with mycobacterial antigens and the quantification of IP-10 by a sandwich ELISA. IP-10 is found to be a biomarker for the diagnosis of TB in African buffalo [85–87], warthog [89] and wild meerkat [90]. Most of the studies were conducted in African buffalo using bovine IP-10 antibodies (capture and detection antibody) either by a conventional ELISA (using bPPD or PC-EC or PC-HP) or by mQFT system (ESAT-6, CFP-10, TB7). The test has an excellent agreement with IGRA and it is reported to provide more diagnostic accuracy in comparison to conventional IGRA in African buffalo [86, 88]. In addition, it has high thermal stability that would facilitate the heat-inactivation of plasma pathogens and for the safe transport of diagnostic samples [87]. Preliminary studies in warthog and wild meerkat show IP-10 assay as a promising method, but the Se was low in warthog [89, 90].

###### Lymphocyte stimulation test (LST)

This test measures the reactivity of blood lymphocytes to mycobacterial antigens. The PBMCs are stimulated by mycobacterial antigens along with controls (Concanavalin A as positive control and fetal bovine serum or unstimulated medium as negative control) followed by the addition of [^3^H]-thymidine and incubation for 4 days. Lymphocyte stimulation is assessed by the uptake of [^3^H]-thymidine and data are expressed in counts per minute (cpm). Usually, the difference in the OD of antigens with negative control and aPPD is taken into consideration for interpreting the results. The test has been performed in deer [68, 91], badger [92], possum [16] and elephant [65]. The use of MPB70 in red deer resulted in improved Sp, but low Se compared to LST using bPPD [68]. In badger, Se of LST was high, but Sp was low in comparison to ELISA [92]. Lymphocyte stimulation responses were detected in 93% of experimentally infected possums [16]. This method is complicated to perform in field conditions for screening large number of samples because of the time requirement and logistics. Hence, nowadays, it is not usually used for diagnostic purpose.

###### Quantitative reverse-transcription PCR assay (qRT-PCR)

The production of cytokines can be detected by measuring the relative level of gene expression using qRT-PCR. The assay includes reverse transcription of mRNA of cytokines of interest (IL-2, IL-4, IL-10, IL-12p40, IFN-γ, TNF-α) into cDNA, followed by real-time PCR using species specific or cross reactive primers. The relative gene expression of IFNγ in response to bPPD and specific antigens was found to be high in infected red deer, elk [84] or badger [93]. qRT-PCR can be used as an alternative to the current serological methods of diagnosis like the brock test in badger [93]. In red deer and elk, qRT-PCR is found to be superior to LST and Cervigam assay [84]. The assay can be exploited as a major diagnostic platform for use in wildlife, since primers are easier to develop as well as it is a simple, rapid, and sensitive measure of antigen-specific CMI and it does not need double handling of animals as in the case of skin test. The demerits include the need of quick processing of samples and requirement of costly reagents [84, 93].

##### Antibody based tests

Antibody assays are convenient to perform as samples can be stored for prolonged time before processing. They ensure large scale screening of samples obtained ante-mortem and post-mortem and are able to diagnose the progressive disease [94]. In this regard, the Se increases in advanced stages of the disease.

###### Enzyme-linked immunosorbent assay (ELISA)

ELISA is the serodiagnostic technique most extensively used in wildlife. It detects circulating antibodies against MTC, and it is well suitable for large scale screening of the disease, both ante-mortem and post-mortem. There are reports of many in house ELISAs (indirect ELISA of bPPD/P22/LAM/*M. bovis* culture filtrate (MBCF)/MPB83 and MPB70 in red deer, wild boar, warthog, African buffalo, elephant, possum and badger) or commercial ELISA kits (ELISA TB-VK, ELISA-INgezim TB porcine and INgezim Tuberculosis DR in wild boar and warthog and Idexx ELISA in wild bovids) using species specific anti-IgG antibodies or cross reactive antibodies like protein G (as conjugate) available for use in wildlife with varying levels of diagnostic accuracy [24, 25, 95–98] (see Tables 1, 2, 3, 4, 5, 6, 7 and 8). Among them, ELISA is the prime choice for TB diagnosis in swine [25], being bPPD the most common antigen used [24, 25, 68, 97, 98], but with low Sp due to cross reactivity with other non-tuberculous mycobacteria. Serum diluted in skimmed milk supplemented with aPPD (competitive ELISA) was found to have more Sp in badger compared to the simple indirect ELISA [99]. Many other specific or purified antigens (LAM, MPB83, MPB70, ESAT-6 and CFP-10, and P22) have been tested to improve the Sp in wild boar [25], red deer [24, 68], wild bovids [7], badger [99] and possum [100]. However, consequently, in some studies the Se is compromised [80]. Brock test is an indirect ELISA based on MPB83 used for TB diagnosis in badger, which provided high Sp, but low Se [92, 96, 101]. Dachs TB-ELISA, also based on MPB83, has high Se, similar Sp and high predictive values compared to Brock test [95]. Nevertheless, in some other reports, similar or even high Se was achieved with specific or purified antigens in red deer and wild boar [24, 25]. The use of antigenic cocktails (MPB70, MPB83), ethanol extract of *M. bovis* antigen (EVELISA) and prior pre-absorption of *M. avium* subspecies *paratuberculosis* antibodies or antibodies to other environmental mycobacteria as *M. phlei* have also proven promising results in red deer [102]. In wild bovids, Se was low in Idexx ELISA which might be due to the limited sample size used. Moreover, TB is a slow and progressive chronic disease so that antibodies might be detected in the terminal stages only (i.e. buffaloes) [7]. In elephants, substantial humoral immune response was detected in positive animals which could help in differentiating positive and negative animals [65, 98]. In possum, low Se was observed in antibody mediated diagnosis, possibly due to the fact that very few possums with sub-clinical *M. bovis* infection produce positive antibody responses [100].

###### Fluorescence polarization assay (FPA)

This test comprises the use of the target antigen MPB70 with a fluorescent molecule bound to it, in order to detect antibodies in serum. This assay was first described by Surujballi et al. (2002) in cattle [103], and later it has been validated in red deer [91], elk [104] and wild bison [105], but with comparatively low diagnostic value in wild bison and cervids when the test is used alone [104, 105]. The Se is very low in early stages of infection [75].

###### Multiantigen print immunoassay (MAPIA)

The MAPIA uses a panel of 12 mycobacterial antigens including eight purified recombinant proteins (ESAT-6, CFP-10, MPB64, MPB59, MPB70, MPB83, Acr1, and the 38 kDa protein), two protein fusions (CFP-10/ESAT-6 and Acr1/MPB83), and two native antigens, such as bPPD and MBCF [106]. The assay enables the qualitative identification of species-specific immunodominant proteins as well as the reactivity patterns over the course of the disease which, in turn, helps in the selection of antigens for other diagnostic tests [106]. The MPB83 alone or in combination with the protein Acr1 was found to be the most serodominant antigen followed by ESAT-6/CFP-10 in elk and fallow deer [107, 108], red deer-elk hybrids [75], white-tailed deer [108, 109] and possum [109]. MPB83 is also the most serodominant antigen in wild boar and warthogs [109, 110]. In badger, MPB83 and MBCF are the most serodominant antigens [101, 109] followed by MPB70 [101] or CFP-10/ESAT-6 [109]. ESAT-6 alone is the most serodominant protein in wild bison [111], while ESAT-6 and CFP-10 are in elephant [10, 108] and black rhinoceros [112]. The MAPIA gave almost equal or even high Se and Sp in comparison to other rapid tests, ELISA or immunoblot, but there is a practical difficulty to implement this assay for screening large number of samples [55, 101].

###### Immunoblotting (IB)

Electrophoresis and immunoblot are qualitative assays, usually performed using whole cell sonicate antigen in reindeer [55], white-tailed deer [113] and elephant [48], but it is not routinely used for diagnosis in any species. Instead, the test is used to confirm that the real antibodies are detected in other serodiagnostic tests like ELISA/MAPIA/immunochromatographic test.

###### Lateral flow tests

These tests are based on the immunochromatography. Lateral flow test kits have great practical applicability in wildlife because of its easiness to perform and immediate test results, although their Se is limited. Most of the lateral flow tests are qualitative except DPP test. The main lateral flow diagnostic tests are listed below:

- *TB STAT-PAK* (Chembio Diagnostic Systems, Inc., Medford, NY)**.** This test employs a unique cocktail of MPB83, ESAT-6 and CFP10 antigens, with a single-strip bead-based signal detection system [75]. The use of this test has been reported in multiple species, i.e. Cervid TB STAT PAK in deer [75], Brock TB STAT-PAK in badger [96], Bovid TB STAT PAK in African buffalo [114] and wild meerkat [9], TB STAT PAK in pygmy hippopotamus [68] and elephant TB STAT PAK in elephant [10], lion [115], black rhinoceros [112] and banded mangoose [116]. Its advantages include easiness to perform in field with a small volume of blood, serum or plasma, and possibility to detect immunoglobulin (Ig) A (IgA), IgM and IgG antibodies to MTC [117]. The test has also legitimate diagnostic accuracy in most of the species tested. However, the performance of test was poor in African buffalo as in the case of ELISA [114] and showed poor Se in wild meerkat when the test was used alone [9]. In comparison to DPP test, false positive results can occur due to the presence of non-tuberculous mycobacteria or inflammatory conditions in elephant [118], as well as false negative results may happen due to the limited Se [52].
- *DPP tests* (Chembio Diagnostic Systems, Inc., Medford, NY). This assay has two test antigen bands on the membrane strip, T1 (MPB83 protein) and T2 (CFP-10/ESAT-6 fusion), for differential IgG antibody detection by colloidal gold particles coupled with hybrid protein A/G, in contrast to the single-strip format used in the TB STAT-PAK test [10]. One of the DPP tests is DPP Vet TB in which the presence and intensity of either of the two separate test lines (MPB83 antigen and CFP-10/ESAT-6 fusion protein) are evaluated visually and by a DPP optical reader [10]. The DPP Vet TB has been employed in multiple species of deer [119–121], wild suids [110], elephant [10, 122] and badger [123]. The DPP Vet TB has higher Sp than STAT PAK in elephant [10] and deer [119]. The DPP VetTB assay is approved by the United States Department of Agriculture (USDA) Bovine TB Eradication Program for testing several species of captive cervids [108, 121] and it is considered appropriate for use in a badger bovine TB control campaign in Northern Ireland [123]. Another test based on DPP technology is DPP WTB which makes use of two antigens in separate test lines (MPB83 and MPB70). This assay is mainly focused for diagnosis in suidae, since MPB70 antigen is more serodominant than CFP-10/ESAT-6 in suids and it is found to be more sensitive than DPP Vet TB in wild boar [124]. The DPP bovid TB is another kind of test based on CFP-10/ESAT-6 and MPB70/MPB83 chimeric antigens, but it had low Se in wild bovids which is consistent with reports of other serological assays in wild bovids.
- *INgezim TB-CROM Ab* (INGENASA S.A., Madrid, Spain). This is a recently developed test in which INgezim TB-CROM Ab uses MPB83 antigen. In wild boar, test acquired high diagnostic value, as well as concordance with ELISA (in house ELISA, commercial ELISAs- INgezim TB porcine**,** INgezim Tuberculosis DR) so that INgezim TB-CROM Ab can be used as a first approach for the surveillance of TB in this species [125].

##### Other tests

Blood tuberculosis test (BTB) is a composite test, comprising of ELISA reactivity towards mycobacterial antigens as well as lymphocyte stimulation [65]. The test has been employed in red deer [126] and elephant [65]. However, this test is no longer in use because of its complexity to perform, especially in elephants, in which multiple tests can delay the treatment protocol [65]. Another biomarker, monocyte chemoattractant protein (MCP)-1, could also be a sensitive marker for TB diagnosis in African buffalo [85]. Gene expression assay of the chemokine (C-XC motif) ligand 9 (CXCL9) is a useful tool for the determination of *M. bovis* status in free-ranging lions [127]. Analysis of volatile organic compounds (VOCs) obtained from breath and feces by electronic nose or gas chromatography–mass spectrometry (GC–MS) is a promising tool for noninvasive detection of TB in badger [128], white-tailed deer [129] and wild boar [130].

### Confounding factors

There are many factors related to host, environment, sampling and diagnosis technique which can affect the performance of the TB diagnostic test:

#### Host

Reports are available about the effect of age and sex on diagnostic accuracy in some species. There are no evident age-related differences in red deer and fallow deer in the responsiveness to skin test [21]. Males had greater response than females in skin test in cervids and this gender based difference was more evident with increasing age [21]. This difference could be due to the differences in reproductive effort and energy expenditure [131]. Moreover, males tend to have a thicker skin than females, so skin fold increase is related to the thickness of the skin in red deer [132] and fallow deer [21]. In wild boar, an increase in skin responsiveness with age was noticed, but there was no sex by age interaction [22]. Serodiagnostic techniques in white-tailed deer showed no age or sex related differences (Immunoblot, MAPIA, ELISA, CervidTB STAT-PAK) [113]. In wild boar, infected piglets had lower Se in ELISA and DPP tests, as compared to yearling, juvenile or adult wild boar [124, 125]. Gender-based variation was not significant in serodiagnostic tests like ELISA and DPP test in wild boar [133]. In badger, Se of IGRA was low in cubs compared to adults, but Sp of IGRA, Brock test and Brock TB STAT-PAK was high in cubs compared to adults [96]. The immunological status of the host needs to be considered, as anergic animals do not respond to diagnostic tests [23].

#### Environment, habitat and management

Skin responsiveness to mitogen in winter was found to be significantly higher compared to the response in summer and the difference was more prominent in adult red deer [132]. This may be due to seasonal presence of non-tuberculous mycobacteria, which is a main confounding factor in TB diagnosis in all species, especially in ruminants like deer [77] and wild bovids [134]. Sp of the serological assays varied between badger populations/countries of origin (higher Sp in badgers of Spain i.e. 96.88–100% compared to those of Republic of Ireland i.e. 85.7%) possibly as a result of variable exposure to different environmental mycobacteria species [99]. Also, the type of management practices which determine the exposure to non-tuberculous mycobacteria could be another factor resulting in the variation of Sp (high Sp in intensive management compared with extensive management in suids) [25].

#### Prior sensitization, history and other infections

The preliminary skin testing for TB can also lead to the diagnosis of false positive animals with other diagnostic techniques [126]. Se of ELISA was considerably increased in red deer 10 days after skin test compared to the Se before skin test (45.7% to 85.3% in ELISA) [126]. In experimental infection in white-tailed deer, reindeer, red deer and black rhinoceros, an elevated antibody response (in ELISA, immunoblot analysis, MAPIA and rapid tests) could be detected shortly after skin testing [55, 75, 112, 119]. However, assays of repeated comparative skin testing in red deer at 6 months interval confirmed that it did not affect serological results [78] and lower IFNγ response was detected in TB positive African buffalo after skin test [66]. On the other hand, past exposure to MTC/BCG vaccination can result in false positive reactions in red deer [135], whereas cross reactions with other non-tuberculous mycobacteria could be a factor affecting the Sp of both cell mediated and humoral tests in several species [24, 77].

#### Sample and sampling related factors

The in vitro production of IFNγ in IGRA and IGRA ELISPOT is influenced by the blood storage temperature and duration of storage until processing (recommended storage at room temperature and maximum time for processing is 8 h), as well as by the type of anticoagulant used (optimal response with heparin) [52, 72, 80]. CMI based tests require fast processing of the sample, while appropriately stored samples can be used in antibody based diagnosis. CMI response measured in vitro is significantly reduced in blood taken immediately after death of the animal [80]. However, overnight stored blood samples had improved Sp in LST in badger without any change in Se (Sp 100% in samples stored overnight and 84.6% in fresh samples) [92]. Incubation of plasma at 65 °C for 20 min or plasma stored on Protein Saver Cards for 2 and 8 weeks did not cause any considerable loss of IP-10 concentration in IP-10 assay which allows the short term storage and transport of samples [87]. In a similar way there was no change in the IFNγ production in samples in which maintenance media (RPMI-1640 medium containing fetal calf serum l-glutamine and penicillin–streptomycin) was added and stored at 4 °C [72]. For humoral-based tests, the antibody responses varied between the type of the sample, i.e. serum, plasma, fresh whole blood, diaphragm fluid and aqueous humor [109, 123]. The source of the sample can also cause variation in diagnostic results, observing that serological test results for hunter or veterinarian harvested blood samples had higher level of agreement with culture results than samples from carcasses with TBL [113]. Repeated freeze-thawing cycles, delay in shipping and occurrence of haemolysis could possibly affect the results of these serological tests [48]. Thus, high levels of haemolysis decreased the Se of antibody tests, being more evident for the bPPD ELISA, but not affecting the results of rapid tests [113], which are designed not only for serum, but also for whole blood.

#### Diagnostic technique related factors

In all methodologies of TB diagnosis, the Se and Sp greatly depends on the type of antigen used, improving the diagnostic accuracy through the use of specific or purified antigens (see Tables 1, 2, 3, 4, 5, 6, 7 and 8). In skin test, there can be technical variation in results depending on the site of inoculation, dose and potency of tuberculin administered and reproducibility between operators [76]. There can be variation in Se depending on the type of assay used to measure the IFNγ released (ELISA/ELISPOT) [136] or the use of monoclonal pairs (mEIA) or polyclonal antiserum (pEIA) against IFNγ (80.9% Se for mEIA, 74.5% for pEIA) [80]. In the humoral diagnostic tests, the conjugate used could also influence Se, obtaining better performance in ELISA with species-specific conjugate (anti-IgG pig- 46.2% Se) than with protein G conjugate (23.1% Se) in wild boar [124]. In quantitative tests, their interpretation depends on the selected cut off value which, in turn, depends on the preferred outcome of the test (better Se or Sp or better compromise between Se and Sp) because Se increases at the expense of Sp and vice versa [81, 137].

Overall, reference tests used have a major influence on the validated diagnostic tests. The gold standard test, mycobacterial culture, has been widely used in validation; however, it can have variable Se and heavy reliance on the number and quality of tissues examined at necropsy [104, 133]. In some studies, skin test or presence of TBL or IGRA or STAT PAK or histopathology were used alone or in combination as reference standards for deer [68, 120, 138], badger [139] and wood bison [111]. In case of wildlife, culture of samples has little practical applicability as far as the difficulty in collection and processing are concerned. Hence, now culture has been considered as an imperfect gold standard for validation of new diagnostic tests in wildlife, as it may underestimate the Se and Sp of newer diagnostic tests being validated [9, 104]. In this regard, Bayesian analytical technique is proved to be a good alternative for estimating the diagnostic accuracy of tests [104, 105].

### Improved diagnosis

#### Selection of the appropriate test

The selection of an appropriate test is based on many factors like species being tested, stage of the disease, diagnostic accuracy of the test, economic feasibility, ease of performing the test, as well as purpose of diagnosis. Skin test is usually employed in deer and wild bovids [63, 68], but it has limited application in other species like suidae, elephant, badger, possum or lion [22, 62, 65, 74]. The stage of the disease progression is a major factor involved in the selection of a test, being CMI-based tests more accurate in identifying the early stages of infection in contrast to antibody-based tests which are more useful in later stages than in early ones [23, 24]. Moreover, in the antibody tests, the antigen MPB83 is detected early in the course of experimental MTC infections [55], unlike MPB70, which elicits a humoral response to MTC in the later stages of the disease [140]. On the other hand, CMI tests, especially skin test, IGRA and IGRA ELISPOT can only be applied for the diagnosis of live animals, while antibody-based can be applied to both live and dead animals [25]. Among the latter, the ELISA techniques are useful for evaluating a large number of samples [25] whereas rapid tests are easy to perform (also in field conditions) and give rapid results compared to any other test [119]. However, rapid tests are not economically viable for screening large number of field samples. In addition to all, it is important to consider the objective intended to choose a diagnostic test for the detection of TB, so a test with high Se is required when the diagnosis is performed for detection of the maximum number of positive animals [104], while a high Sp is the major factor when the test is done for test and slaughter procedures in order to avoid false positive reactors and thereby a huge economic loss [77].

#### Proper implementation and interpretation of the test

Proper implementation of the test procedure is especially important to minimize the diagnostic errors and for better results. The Se and Sp are the major factors evaluated for assessing the test result, being a highly sensitive test usually related to a low Sp and vice versa [137]. Thus, a higher cut off value minimizes the chance of false positives, but lower cut off values enable the maximum identification of infected animals [99, 137]. In addition, predictive values, likelihood ratio and diagnostic odds ratio have importance in interpreting the results [70]. The prevalence of disease must be taken into account for interpreting the result, since a higher prevalence tends to lead to an increased positive predictive value (PPV) and a decreased negative predictive value (NPV), whilst a lower prevalence tends to lead to an increased NPV and a decreased PPV [141].

#### Combination of different diagnostic tests or different antigens

Combination of two diagnostic test results in parallel [40, 71, 104, 142] or in series [21, 142] can enhance the diagnostic accuracy. Parallel testing is a method in which two screening tests are performed at the same time and the results are subsequently combined, resulting in higher Se but lower Sp. Serial testing means that both techniques can be performed sequentially; if the result of the first screening test is positive (serial testing), the second screening test will be performed to avoid false positive results [104]. Usually, CMI-based tests are interpreted in parallel to antibody-based tests resulting in very high Se which aids to detect a maximum number of animals in different stages of the disease, thereby facilitating test and removal strategies for the disease control in wildlife. Thus, the combination of Bovigam assay (bPPD antigen) and SCITT/IGRA/Idexx ELISA or Bovigam assay (bPPD, PC-EC, PC-HP) yielded 100% Se in parallel interpretation in African buffalo, while the second Bovigam assay alone offered a Se of 75% [7]. Similarly, the parallel use of SCITT with Bovigam assay or IP-10 assay was also able to identify all the infected animals in African buffalo [71]. In the same way, parallel interpretation of SCITT and Cervid TB STAT-PAK allowed the detection of all *M. bovis* confirmed by culture in fallow deer [21], and the parallel use of FPA, LST and Cervid TB STAT-PAK identified all the infected elk [104]. Pathological lesions and culture results were interpreted in series to determine true infection status in fallow deer for minimizing the number of false positives [104]. Combination of Brock TB STAT PAK with IGRA and culture allowed a diagnosis to be made for individual animals with an estimated overall accuracy of 93% in badger [142, 143].

The use of multiple antigens in the same diagnostic test (IGRA, ELISA or rapid tests) and its further interpretation improved the diagnostic accuracy. In red deer, the evaluation in parallel of ESAT-6/CFP-10 with Rv3615c and Rv3020c antigens increased the Se of the technique when compared to the separate use of these antigens [23]. Moreover, multi-antigen ELISA also enhanced Se of TB diagnosis in badger [95] and elephant [65, 98].

## Conclusions

**Figure 3:**
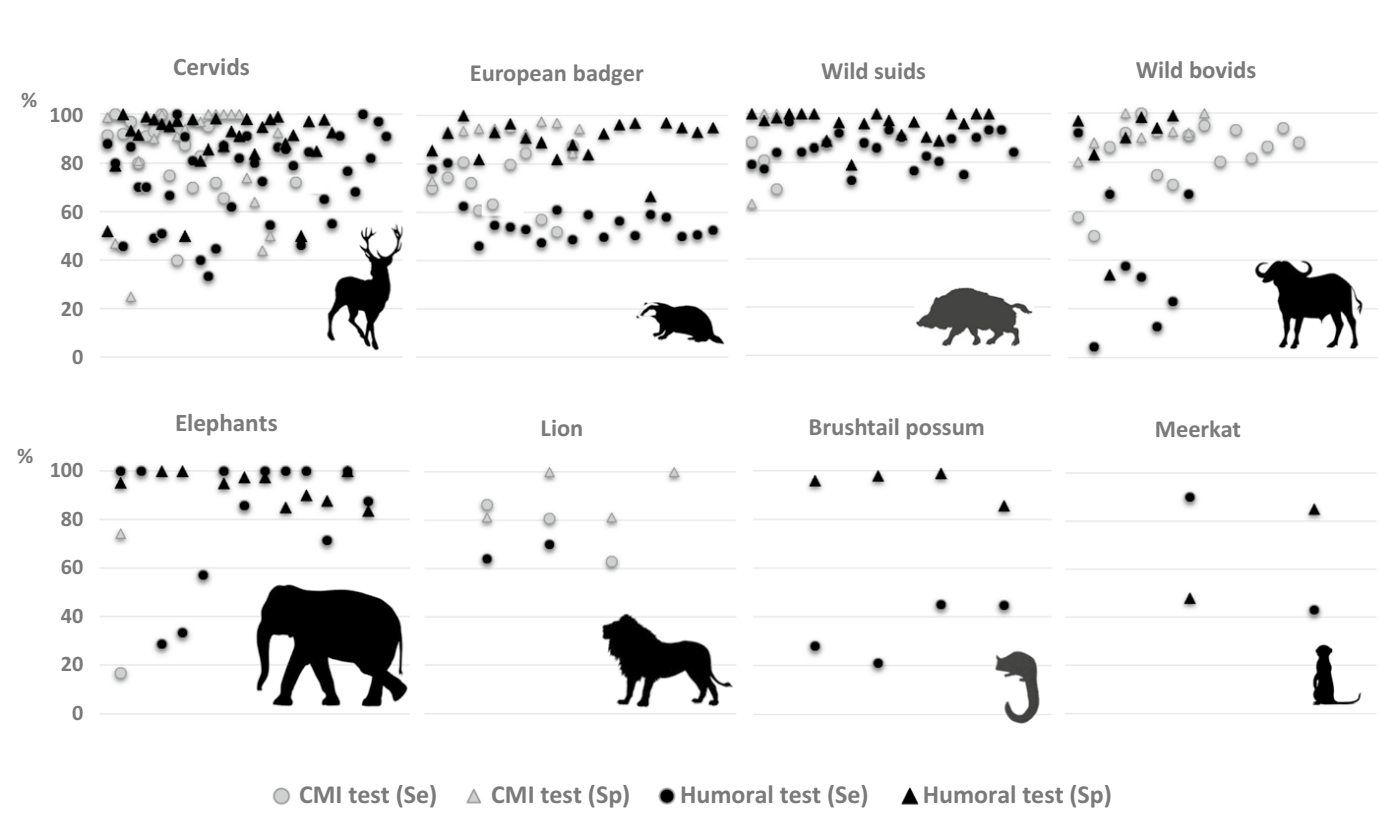
**Sensitivity (Se) and specificity (Sp) of immunological tests used in wildlife tuberculosis diagnosis in cervids (n = 108 evaluations), European badger (n = 59), wild suids (n = 48), wild bovids (n = 39), elephants (n = 28), lion (n = 9), brushtail possum (n = 8) and meerkat (n = 4).** Details can be consulted in Tables 1, 2, 3, 4, 5, 6, 7 and 8. CMI: diagnostic methods based on cell-mediated immunity.

Culture and identification of MTC remain as the gold standard method in wildlife TB diagnosis, even so limited Se and little practical application exists as far as the difficulty in collection and processing of samples are concerned. In this regard, pathological studies can help to increase the culture Se. In recent years there has been considerable progress in wildlife TB diagnosis, where cellular and humoral immunological diagnostic tests are gaining importance, mainly in cervids, badgers, wild bovids and wild suids (Figure 3). Regarding cellular based diagnostic techniques, SIT is the official ante-mortem test in many species, especially in cervids; however, the development of IGRA offers an improvement in the diagnostic accuracy not only in cervids, but also in African buffalo or badgers. Serological tests are especially useful in wildlife because they are economically attractive, technically easy, the large-scale surveillance is possible in a short period of time and tests can be applied either in live or dead animals. Lateral flow tests are very convenient for use in wildlife since they are effortless to perform and give rapid results; however, Se and Sp of these tests must be still improved. Variability in the Se and Sp of the same technique has been observed according to the target species (i.e. SIT in cervids and badgers) and, therefore, the testing strategy should be also adapted to the target species, as well as to the logistic and budget constraints. According to the information collected, serological tests for MTC-antibody detection are especially important in wildlife, since the possibility of being used together with the post-mortem examination supposes a sensitive and cost-effective means of disease surveillance that should be maintained or implemented; while tests based on CMI are still relevant in wild ruminants. In farmed or easily handled wild animals, it is important to highlight that combinations of cellular and humoral tests could enhance the diagnostic accuracy, since animals in different stages of the disease would be detected. Future studies are still needed in the area of wildlife TB diagnosis in order to reach an accurate, rapid and cost-effective diagnosis in target species. Moreover, testing must be consistent over space and time to allow proper disease monitoring.

## Supplementary Information

**Additional file 1.** **Search algorithms used for collection of articles. **Different algorithms used in collection of data for systematic review are explained.

**Additional file 2.** **An overview of tuberculosis (TB) diagnostic tests in wildlife**.

## Data Availability

The data and materials will be available upon request.

## Abbreviations

aPPD: Avian purified protein derivative
BAL: Bronchoalveolar lavage
BCG: Bacillus calmette guerin
bPPD: Bovine purified protein derivative
BTB: Blood tuberculosis test
CFP-10: Culture filtrate protein-10 kDa
CFU: Colony forming unit
CMI: Cell-mediated immunity
DIVA: Differentiation of infected and vaccinated animals
DPP: Dual path platform
ELISA: Enzyme-linked immunosorbent assay
ELISPOT: Enzyme-linked immunospot assay
ESAT-6: Early secretory antigenic target-6 kDa
FPA: Fluorescence polarization assay
IB: Immunoblotting
IFNγ: Interferon gamma
Ig: Immunoglobulin
IGRA: IFNγ response assay
IL: Interleukin
IP-10: IFNγ-inducible protein 10
LAM: Lipoarabinomannan
LN: Lymph node
LST: Lymphocyte stimulation assay
MAC: *Mycobacterium avium* complex
MAPIA: Multiantigen print immunoassay
MBCF: *Mycobacterium bovis* culture filtrate
MPB 83: Mobility protein of *M. bovis* 83
MPB 70: Mobility protein of *M. bovis* 70
mQFT: Modified QuantiFERON^®^ TB gold in-tube
MTC: *Mycobacterium tuberculosis* complex
NPV: Negative predictive value
OD: Optical density
OIE: Office international des epizooties
PBMC: Peripheral blood mononuclear cells
PBS: Phosphate buffered saline
PCR: Polymerase chain reaction
PPV: Positive predictive value
PRISMA: Preferred reporting items of systematic reviews and meta-analyses
qRT-PCR: Quantitative reverse-transcription polymerase chain reaction assay
RPMI: Roswell Park Memorial Institute medium
SCITT: Comparative intradermal tuberculin skin test
Se: Sensitivity
SIT: Single intradermal tuberculin skin test
Sp: Specificity
TB: Tuberculosis
TBL: TB like lesions
