# Supplementary material for: Diagnosis of tuberculosis in wildlife: a systematic review
Source: Vet Res. 2021 Feb 24;52:31. doi: 10.1186/s13567-020-00881-y (PMC7905575; doi:10.1186/s13567-020-00881-y)
Supplement: Supplementary file 1 — Additional file 1. Search algorithms used for collection of articles. Different algorithms used in collection of data for systematic review are explained. [file 13567_2020_881_MOESM1_ESM.rtf]

Additional file 1: Search algorithms used for collection of articles.

Ø	 (Wildlife OR wild animal) AND (tuberculosis) AND (diagnosis OR test OR detection OR identification).
Ø	(Deer OR elk) AND (tuberculosis) AND (diagnosis OR test OR detection OR identification).
Ø	(Wild boar OR warthog OR feral pig OR bush pig OR collared peccaries) AND (tuberculosis) AND (diagnosis OR test OR detection OR identification).
Ø	(Buffalo OR African buffalo OR wood bison OR tapir OR nyala) AND (tuberculosis) AND (diagnosis OR test OR detection OR identification).
Ø	(Badger) AND (tuberculosis) AND (diagnosis OR test OR detection OR identification).
Ø	(Possum) AND (tuberculosis) AND (diagnosis OR test OR detection OR identification).
Ø	(Wild meerkat OR mangoose) AND (tuberculosis) AND (diagnosis OR test OR detection OR identification).
Ø	(Elephant OR rhinoceros OR hippopotamus OR lion OR red fox OR giraffe) AND (tuberculosis) AND (diagnosis OR test OR detection OR identification).
