# Supplementary material for: Diagnosis of tuberculosis in wildlife: a systematic review
Source: Vet Res. 2021 Feb 24;52:31. doi: 10.1186/s13567-020-00881-y (PMC7905575; doi:10.1186/s13567-020-00881-y)
Supplement: Supplementary file 2 — Additional file 2. An overview of tuberculosis (TB) diagnostic tests in wildlife. [file 13567_2020_881_MOESM2_ESM.rtf]

Additional file 2. An overview of tuberculosis (TB) diagnostic tests in wildlife. 
Test	Remarks	Constraints	
TBL	Detection of TBL macroscopically by PM study and microscopically by histopathology. PM study remains as a sensitive and cost-effective means of disease surveillance. Histopathology is also not 100% confirmatory, complementary diagnostic techniques are needed for confirmation. 	Lesions are noticeable in advanced stage, lesions in non-classical sites makes diagnosis difficult, indistinguishable macroscopic lesions are also seen in other infections. 	
Culture	Gold standard method for the diagnosis of TB, live sampling from BAL/ tracheal washing or PM sampling from organs with TBL followed by culturing in specific media. Identification of the microorganism after cultural isolation can be done by colony characteristics, biochemical tests or nucleic acid recognition methods. 	Expensive, time-consuming and requires biosafety level 3 laboratory, variable Se due to absence of organism in collected sample, stage of disease, sample quality etc.,  decontamination step prior to culture can affect the viability of mycobacteria.	
Molecular methods	Nucleic acid recognition methods like simple PCR/ genotyping, rapid and highly sensitive method, useful for epidemiological studies, helpful to reach immediate control decisions in some species.	Variable Se due to absence of organism in collected sample.  Requires costly reagents/ equipment like primers, DNA polymerase, thermal cycler etc.	
Skin test	In vivo stimulation of animal with mycobacterial antigens and measuring the skin thickness after 72 hours. Not practical in wildlife. Useful in captive wildlife and zoo animals. Official ante-mortem test in cervidae and bovidae. Less relevant in other wildlife species.	Difficulty in handling animals twice and stress associated with double handling, technical variability, low Sp.	
IGRA	Alternative or supplementary assay to the skin test, in vitro stimulation of PBMCs to produce IFNã and detection of IFNã by sandwich ELISA. Requires single handling of animals. Use of specific antigens improves the Sp. Main issue is the loss of Se when using specific antigens. New diagnostic platforms like mQFT system provides better Sp without compromising Se in African buffalo. Preliminary studies performed in red deer, badger, warthog and rhinoceros with specific/purified antigens are promising. 	Need of fast processing of samples, strict laboratory conditions, reagents like species specific IFNã antibodies, mycobacterial antigens, controls, medium etc and equipment like ELISA reader, incubator etc., expensive when compared to skin test. 	
IGRA ELISPOT
	Similar to IGRA ELISA, but detecting IFNã by ELISPOT. Requires single handling of animals and Sp improves when using specific antigens. Studies are conducted only in badger with high diagnostic accuracy.	 Need for fast processing of samples, difficulty in collecting samples from live animals, need of strict laboratory conditions, reagents, equipment and it is expensive as in case of IGRA. Lack of studies in other species.  	
IP-10 assay	Stimulation of whole blood with mycobacterial antigens and quantification of IP-10 by a sandwich ELISA. Single handling of animals, Sp improves when using specific antigens, high thermal stability and IP-10 can be measured on plasma stored on Protein Saver Cards for short periods of time. Extensive studies in African buffalo resulted in high diagnostic accuracy in this species and low Se in warthog. Preliminary study conducted in wild meerkat is promising.	Need of expensive reagents (anti IP-10 antibodies and other reagents in IGRA) and rigid laboratory conditions as in the case of IGRA. Lack of studies in many wildlife species. 	
qRT-PCR of cytokines expression 	Relative gene expression of cytokines in response to in vitro stimulation by mycobacterial antigens. Simple, rapid and sensitive measure of antigen-specific CMI. IFNã qRT-PCR has been tested in badger, red deer and elk, being a useful diagnostic platform for wildlife.	Expensive reagents (anti-cytokine antibodies, PCR reagents), requirement of equipment (thermal cycler) and need of quick processing of samples.	
LST	Reactivity of blood lymphocytes in response to in vitro stimulation by mycobacterial antigens by assessing the uptake of [3H]-thymidine. Tested in badger, possum, red deer and elephant with appreciable diagnostic accuracy.	Complicated in terms of time requirement and logistics. Not suitable for screening large number of samples.	
ELISA	Quantitative detection of circulating antibodies against MTC by using mycobacterial antigens in ELISA. Useful in advanced stages of the disease. Prime choice of diagnosis in suids. In house and commercial ELISAs have been developed for multiple species. Usually high Se and low Sp when using common antigens, but low Se and high Sp when using specific/purified antigens.	Cross-reactivity when using bPPD as antigen. Commercial ELISA kits are expensive. Not reliable in early stages of disease.	
FPA	Detection of circulating antibodies against MTC by use of MPB70 bound to a fluorescent molecule. Low diagnostic value in wild bison, red deer and elk. Not evaluated in other species.	Low Se in early stages of disease as in case of other antibody mediated diagnostics. Need of equipment like fluorescent microscope.  	
MAPIA	Qualitative identification of species-specific immunodominant proteins by using a panel of 12 mycobacterial antigens, helps in the selection of antigens for other diagnostic tests in each species. Performed in multiple wildlife species to determine reactivity patterns of different mycobacterial antigens.	Practical difficulty to implement this assay for screening large number of samples. Expensive. 	
IB	Qualitative assay performed as immunoblot is useful for confirming that the real antibodies are detected in other serodiagnostic tests. Performed in reindeer, white-tailed deer and elephant.	Not suitable for screening large number of samples. It is not a routine diagnostic tool.	
Lateral flow tests
	Based on the principle of immunochromatography. Useful in wildlife. Easy to perform and rapid test results. Whole blood and serum samples can be used.  TB STAT-PAK, DPP test, INgezim TB-CROM Ab are lateral flow assays used in several wildlife species.		Lateral flow test kits are expensive. All lateral flow tests are qualitative except DPP assay. Difficult to standardize test kits of appropriate diagnostic accuracy in different species.	

TB: Tuberculosis; TBL: TB like lesions, PM- Post mortem, BAL: Bronchoalveolar lavage; PCR: Polymerase chain reaction; IFNã: Interferon gamma; PBMC: Peripheral blood mononuclear cells; Se: Sensitivity; Sp: Specificity; mQFT: modified QuantiFERON® TB Gold In-Tube; IGRA: IFNã response assay; qRT-PCR: Quantitative reverse-transcription PCR assay; ELISPOT: Enzyme-linked immunospot assay; IP-10: IFNã-inducible protein 10; LST: Lymphocyte stimulation assay; ELISA: Enzyme-linked immunosorbent assay; FPA: Fluorescence polarization assay; MAPIA: Multiantigen print immunoassay; IB: Immunoblotting; bPPD: Bovine purified protein derivative; MTC: Mycobacterium tuberculosis complex. 
